# Activation of *BmToll9-1* in Silkworm (*Bombyx mori*) Larval Midgut by *Escherichia coli* and Regulation of Growth

**DOI:** 10.3390/insects16060621

**Published:** 2025-06-11

**Authors:** Jisheng Liu, Weijian Chen, Minchun Lai, Jiahua Chen, Luc Swevers

**Affiliations:** 1 School of Life Sciences, Guangzhou University, Guangzhou 510006, China; 2 Institute of Biosciences and Applications, National Centre for Scientific Research Demokritos, 15431 Athens, Greece; swevers@bio.demokritos.gr

**Keywords:** *Bombyx mori*, *BmToll9-1*, RNAi, immune response, Gram-negative bacteria, *Escherichia coli*

## Abstract

Insects rely on their innate immune system to defend against pathogens, and the Toll signaling pathway plays an important role in immune regulation. Our previous studies have shown that *BmToll9-1* functions as a positive regulator in the Toll pathway. This study seeks to elucidate the role of *BmToll9-1*, as a sensor to bacterial challenge, in modulating larval development and downstream Toll signaling pathways. Silkworm larvae were subjected to infection with either Gram-negative *Escherichia coli* or Gram-positive *Staphylococcus aureus* bacteria following silencing of *BmToll9-1* by RNA interference (RNAi). This bacterial challenge triggered a compensatory re-induction of *BmToll9-1* expression, which resulted in the recovery of larval weight and size to levels observed in untreated controls. Furthermore, upon bacterial infection of *BmToll9-1*-silenced larvae, there was an up-regulation in the expression of both signaling genes in the Toll pathway and downstream effector genes, with a marked preference for Gram-negative bacteria. These results highlight the involvement of *BmToll9-1* in the Toll signaling pathway as a positive regulator, influencing silkworm development. Additionally, *BmToll9-1* and *BmToll9-2* were cross-validated to be genetically distinct genes, even though they were confirmed to be functionally analogous in the silkworm.

## Simple Summary

Toll receptors play a crucial role in insect development and innate immunity. *BmToll9-1* has been proven to positively regulate both development and immune responses in silkworms. *BmToll9-2* appears to exhibit similar functions. This article further explores the impact of bacterial infection on silkworm larvae following RNA interference of *BmToll9-1*. Interestingly, bacterial infection seems to reactivate the silenced *BmToll9-1* and nullify the phenotypic changes it caused, while also activating downstream immune pathways, with a particularly pronounced response against Gram-negative bacteria (specifically *Escherichia coli*, when compared to Gram-positive *Staphylococcus aureus*). These functional characteristics show a remarkable similarity to those of *BmToll9-2*, which were previously reported. However, a comprehensive cross-validation confirms that they are genetically distinct genes despite their functional parallels.

## 1. Introduction

Insects have flourished on Earth for more than 480 million years and this can be explained, at least partially, by their powerful immunity. Insects primarily defend against the invasion of exogenous pathogens through their innate immune system [1], which synthesizes and secretes antimicrobial peptides (AMPs) mediated by Toll and immune deficiency (IMD) signaling pathways [2]. Additionally, insects can also defend against exogenous pathogens through cellular immunity mediated by hemocytes (e.g., phagocytosis, nodulation, and encapsulation).

The Toll/TLR receptors belong to an ancient family of immune defense-related receptors, with genes encoding the signaling cascade components conserved across all vertebrates, spanning from lower to higher taxa [3]. Insect Toll is a type I transmembrane protein that can be divided into three parts: extracellular, transmembrane, and intracellular regions. The transmembrane region is flanked by an extracellular leucine-rich repeat (LRR) domain for ligand recognition and the cytoplasmic Toll/interleukin-1 receptor (TIR) domain that interacts with downstream molecules such as myeloid differentiation factor 88 (MyD88) [4,5,6].

The earliest discovery of Toll receptors was in studies of embryonic development in *Drosophila melanogaster* [7]. Subsequently, a total of nine Toll receptors were identified in *Drosophila* and they differed in structure and function [8,9,10]. Several studies revealed that the overexpression of *DmToll* and *DmToll9* led to a significant activation of antimicrobial peptide (AMP) genes, whereas *Dm18W* and *DmToll5* were induced to be expressed when the organism was infested with exogenous pathogens [11,12,13,14,15]. Also, *Dm18W* was associated with the development of *Drosophila* follicular cells and salivary glands [16,17,18], while *DmToll8* was associated with the regulation of neuron-specific glycosylation and the development of wing discs in the *Drosophila* embryo [19,20]. In addition to *Drosophila*, Toll receptors have been found in other insects. For example, there are 5 Toll receptors in *Apis mellifera* [21], 11 receptors in *Anopheles gambiae* [22], 9 in *Tribolium castaneum* [23], and 14 in *Bombyx mori* [24].

The domesticated silkworm, *B. mori*, which originated in China, has been cultivated for more than 5000 years ago. The silkworm not only holds significant economic importance in China but also serves as a model organism in Lepidoptera. It was the first lepidopteran insect to have its entire genome sequenced [25,26,27]. Moreover, the silkworm’s susceptibility to pathogen infections during cultivation makes it an excellent model for studying the mechanisms of innate immunity [28,29].

Although there are 14 Toll-related genes in silkworms, current studies are focused on the Toll9 genes that encode two closely related receptors, *BmToll9-1* and *BmToll9-2*. Similarly to *DmToll9*, *BmToll9-1* and *BmToll9-2* are associated with the innate immunity of *B. mori*, as proven in previous research [6,30,31,32,33,34,35]. In previous studies, we found that *BmToll9-1* transcripts were reduced after an injection of lipopolysaccharide (LPS) or double-stranded RNA (dsRNA) into silkworm larvae [31] and that LPS also repressed AMP genes and other immune pathway-related genes in silkworm-derived Bm5 cells overexpressing the *BmToll9-1* receptor [32]. *BmToll9-1* expression was induced after infection with *Escherichia coli* and the fungus *Beauveria bassiana* [30,36], while *BmToll9-2* was induced by *E. coli* and its main cell wall component LPS, as well as *Staphylococcus aureus* and its main cell wall component peptidoglycan (PGN) [34]. In our recent studies, we found that the larvae and cocoons were smaller and lighter after the knockdown of *BmToll9-1*, and that *BmToll9-1* played a role as a positive regulator in the immune response of the Toll signaling pathway to enhance antimicrobial activity against *E. coli* [6]. Similarly to *BmToll9-1*, *BmToll9-2* showed the same effect, i.e., it stimulates the humoral immune response and antibacterial activity [35]. Remarkably, a bacterial challenge following RNAi up-regulated the expression of *BmToll9-2* and significantly mitigated the silkworm weight differences [34].

Although we have already demonstrated that *BmToll9-1* plays a vital role in innate immunity, we have yet to pinpoint the pathways involved and the mechanisms that regulate its function. Previously, we confirmed the role of *BmToll9-1* via double-stranded RNA (dsRNA)-mediated gene silencing [6]. In this follow-up study, a bacterial challenge and RNAi were combined to complement previous research and further elucidate the mechanisms by which *BmToll9-1* functions in the innate immunity of the silkworm.

## 2. Materials and Methods

### 2.1. Insect Rearing and Bacterial Culture

The larvae of *B. mori* “P50” strain were provided by the Guangdong Academy of Agricultural Sciences, Guangzhou, China and reared on fresh mulberry leaves at 25 ± 1 °C, 75 ± 5% relative humidity, and a photoperiod of 12 L:12 D. Silkworm larvae at the 5th instar were selected for sampling. All samples were immediately transferred to RNase-free tubes and stored at −80 °C. The bacteria *E. coli* and *S. aureus*, maintained in our laboratory, were grown overnight in Luria–Bertani (LB) liquid medium at 37 °C and 200 rpm.

### 2.2. RNA Protocol and Bacterial Challenge to Larvae

RNAi was performed as described in our previous report [6]. In brief, the T7 RNA polymerase promoter sequence was added to the primers before PCR amplification to produce the template DNA. A T7 RiboMAX Express RNAi kit (Promega, Madison, WI, USA) was used to synthesize dsRNA. Double-stranded *BmToll9-1* (ds*BmToll9-1*) was injected on Day 1 of the 5th instar larvae, while double-stranded green fluorescent protein (ds*GFP*) was used as a negative control (N = 50 per treatment for each treatment).

Subsequently, ds*BmToll9-1*- and ds*GFP*-treated larvae were provided with mulberry leaf disks of identical size, coated with 10 µL of *E. coli* or *S. aureus* bacterial solution [34,35]. Midgut samples were collected at 6, 12, and 24 h after feeding.

### 2.3. RNA Extraction and cDNA Synthesis

Total RNA was isolated from frozen tissue samples using an RNAiso Plus kit (TaKaRa, Kusatsu, Japan) following the manufacturer’s protocol. Genomic DNA was eliminated through DNase treatment, and first-strand complementary DNA (cDNA) was synthesized utilizing a PrimeScript RT Reagent Kit (Perfect Real Time) (TaKaRa).

### 2.4. Quantitative Real-Time PCR (qRT-PCR)

GoTaq qPCR Master Mix (Promega, Madison, WI, USA) was employed for qRT-PCR amplification, with midgut cDNA from 5th instar larvae as the template. The thermal cycling protocol was executed on a CFX Connect Real-Time System (Bio-Rad, Hercules, CA, USA) as follows: 95 °C for two min, 40 cycles of 95 °C for 15 s, and 57 °C for 30 s. After the thermal cycles, a dissociation step ranging from 65 °C to 95 °C was incorporated. Primer sequences for detecting *BmToll9-1*, *BmToll9-2*, signaling genes, and effector genes are detailed in Table 1. Translation initiation factor 4A (*BmTIF4A*) and translation initiation factor 3 subunit 4 (*BmTIF3s4*) were used as the reference genes [37]. Gene expression normalization was achieved through geometric averaging of the reference genes, and relative expression levels of target genes were calculated using the 2^(−∆∆CT)^ method [38]. All qRT-PCR reactions were conducted with three biological replicates (N = 10 larvae), each accompanied by three technical replicates.

### 2.5. Data Analysis

Statistical analyses were conducted using a two-way ANOVA followed by Student’s *t*-test using SPSS version 26.0. Data visualization and plotting were performed in GraphPad Prism v10.0, with results presented as mean ± standard deviation based on three biological replicates. The mean values of knockdown or inhibition rates were compared to the control groups and converted to a percentage.

## 3. Results

### 3.1. Activation of BmToll9-1 by Bacterial Challenges Following RNAi of BmToll9-1

**Figure 1:**
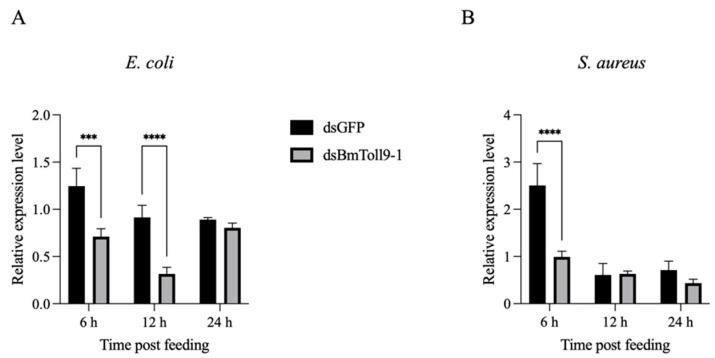
The relative expression of *BmToll9-1* in the midgut of 5th instar larvae injected with ds*BmToll9-1* and fed a diet containing heat-inactivated (**A**) *E. coli* or (**B**) *S. aureus*, compared to the negative control, i.e., larvae treated with ds*GFP*. The data were presented as means ± standard deviations of three biological replicates. The asterisks indicate significant differences from the ds*GFP* injection groups: *** *p* < 0.001, and **** *p* < 0.0001.

In the previous study, *BmToll9-1* could be induced by feeding on *E. coli* and *B. bassiana*, and we confirmed that it was effectively silenced in the midgut via dsRNA [6,30,36]. In this study, to further demonstrate the role of bacterial infections as a trigger for *BmToll9-1* expression, heat-inactivated *E. coli* and *S. aureus* were fed to the silkworm larvae after ds*BmToll9-1* was injected into the larvae. The results showed that the relative expression of *BmToll9-1* in the RNAi group (injected with ds*BmToll9-1*) remained significantly silenced, showing a 43% and 65% reduction after 6 h and 12 h, respectively. However, by 24 h following feeding on food that contained *E. coli*, its expression level had returned to that of the control group (injected with ds*GFP*) (Figure 1A). In contrast, when feeding on food that contained *S. aureus*, the relative expression of *BmToll9-1* in the RNAi group had normalized to the same level as the control group within 12 h (Figure 1B). Therefore, while dsRNA effectively reduced the expression of *BmToll9-1* in *B. mori*, this silencing effect could be mitigated by bacterial infection.

### 3.2. Bacterial Challenges Following Injection of dsBmToll9-1 Mitigated the Diminished Growth Phenotype

**Figure 2:**
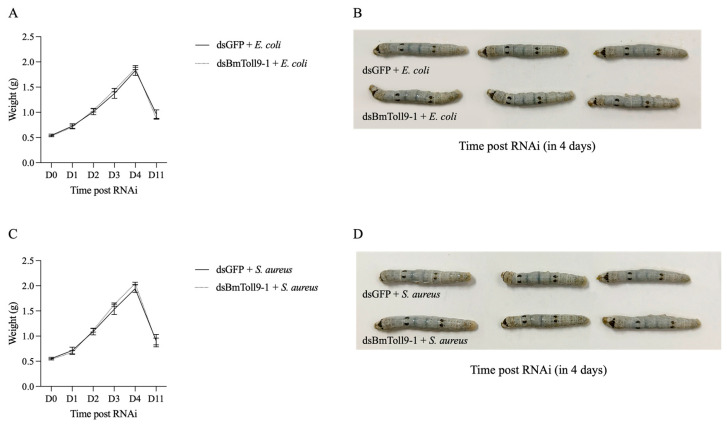
Weight and phenotype changes in *B. mori* 5th instar larvae, following heat-inactivated bacteria feeding treatment after injection with ds*BmToll9-1*, compared to control larvae injected with ds*GFP*. Average weight of silkworm larvae on different days after feeding on (**A**) *E. coli* or (**C**) *S. aureus*, following *BmToll9-1* silencing. Growth phenotype of silkworm larvae on 4th day after feeding on heat-inactivated (**B**) *E. coli* or (**D**) *S. aureus*, following *BmToll9-1* silencing.

After the *BmToll9-1* gene was silenced, both the larvae and cocoons exhibited a smaller and lighter phenotype [6]. However, bacterial treatment annulled the effects of ds*BmToll9-1* injection over time, which manifested as reduced growth, affecting both the body weight and dimensions (Figure 2). Upon feeding on *E. coli*, the body weight of *BmToll9-1*-silenced larvae gradually recovered to match that of the control, reaching a maximum of 1.87 g (on the 4th day after the ds*BmToll9-1* injection) (Figure 2A,C). Both *BmToll9-1*-silenced larvae and the control larvae pupated into the cocoon stage around the same time (the 4th day after the ds*BmToll9-1* injection). After pupation, their body mass decreased to 0.87 g and 0.90 g, respectively, on the 11th day. Similarly, after feeding on *S. aureus*, *BmToll9-1*-silenced larvae showed comparable changes to those treated with *E. coli*, with their body weight gradually returning to the same weight as the control group, peaking on the 4th day post RNAi (ds*BmToll9-1* injection) (Figure 2B,D). On the 11th day after the ds*BmToll9-1* injection, these larvae then entered the cocoon stage, with their body weight reducing to 0.89 g and 0.91 g, respectively.

### 3.3. Bacterial Challenges Following RNAi of BmToll9-1 Induced Signaling Genes in Toll Pathway

**Figure 3:**
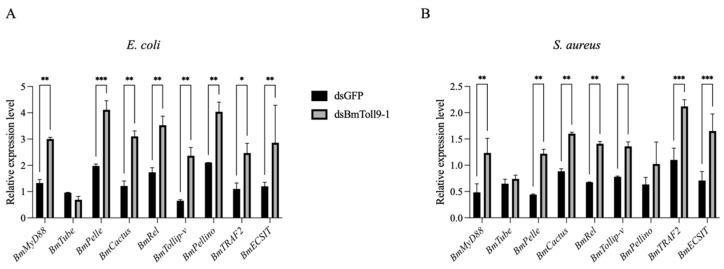
The relative expression of the signaling genes in the Toll pathway in *B. mori* 5th instar larvae, following a heat-inactivated (**A**) *E. coli* or (**B**) *S. aureus* feeding treatment after injection with ds*BmToll9-1*, compared to control larvae injected with ds*GFP*. Data were presented as means ± standard deviations of three biological replicates. Asterisks indicate significant differences towards the ds*GFP* injection groups: * *p* < 0.05, ** *p* < 0.01, and *** *p* < 0.001.

Our previous study confirmed that RNAi of the *BmToll9-1* gene reduced the expression of signaling genes in the Toll pathway and downstream effector genes [6]. To further investigate the role of bacteria, bacterial treatment through the food was administered following the injection of ds*BmToll9-1* to assess the activation of signaling genes. Since both bacterial treatments after RNAi of *BmToll9-1* led to the activation of *BmToll9-1* expression at 24 h, this specific time point was chosen for subsequent validation. Compared to the control group, most of the signaling genes in the Toll pathway were significantly up-regulated in the RNAi group after the bacterial challenges. After feeding on *E. coli*, the expression levels of *BmMyD88*, *BmPelle*, *BmCactus*, *BmRel*, *BmTollip-v*, *BmPellino*, *BmTRAF2*, and *BmECSIT* were induced by 2.26-, 2.08-, 2.55-, 2.03-, 3.64-, 1.92-, 2.25-, and 2.38-fold, respectively (Figure 3A). After feeding on *S. aureus*, the immune genes *BmMyD88*, *BmPelle*, *BmCactus*, *BmRel*, *BmTollip-v*, *BmTRAF2*, and *BmECSIT* were induced by 2.55-, 2.77-, 1.81-, 2.09-, 1.74-, 1.93-, and 2.32-fold, respectively (Figure 3B).

### 3.4. Bacterial Challenges Following RNAi of BmToll9-1 Induced Downstream Effector Genes

**Figure 4:**
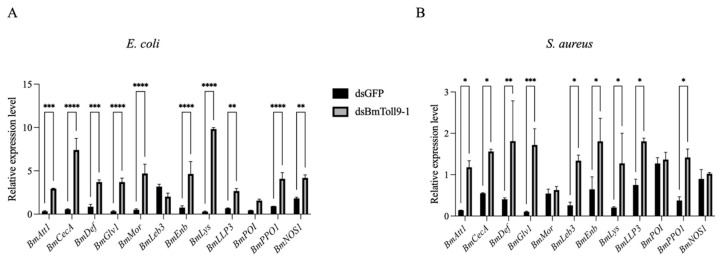
The relative expression of the immune effector genes in *B. mori* 5th instar larvae, following a heat-inactivated (**A**) *E. coli* or (**B**) *S. aureus* feeding treatment after an injection with ds*BmToll9-1*, compared to control larvae injected with ds*GFP*. The data were presented as means ± standard deviations of three biological replicates. The asterisks indicate significant differences towards the ds*GFP* injection groups: * *p* < 0.05, ** *p* < 0.01, *** *p* < 0.001, and **** *p* < 0.0001.

Similarly, bacterial treatment was administered following the RNAi of *BmToll9-1* to evaluate the activation of effector genes (Figure 4). Compared to the control group, most AMP genes were significantly up-regulated in the RNAi ds*BmToll9-1* treated group of larvae after the bacterial challenges. After feeding on *E. coli*, *BmAtt1*, *BmCecA*, *BmDef*, *BmGlv1*, *BmMor*, *BmEnb*, *BmLys*, *BmLLP3*, *BmPPO1*, and *BmNOS1* were significantly induced by 9.06-, 13.23-, 4.36-, 11.31-, 9.21-, 6.15-, 33.29-, 4.02-, 4.36-, and 2.30-fold, respectively, while *BmPOI* was also up-regulated, but not at a statistically significant level (Figure 4A). Following feeding on *S. aureus*, *BmAtt1*, *BmCecA*, *BmDef*, *BmGlv1*, *BmLeb3*, *BmEnb*, *BmLys*, *BmLLP3*, and *BmPPO1* were significantly induced by 8.41-, 2.84-, 4.47-, 16.77-, 5.19-, 2.80-, 6.22-, 2.41-, and 3.77-fold, respectively, while *BmMor*, *BmPOI*, and *BmNOS1* were not induced (Figure 4B).

### 3.5. Silencing BmToll9-1 Did Not Affect the Expression of BmToll9-2

**Figure 5:**
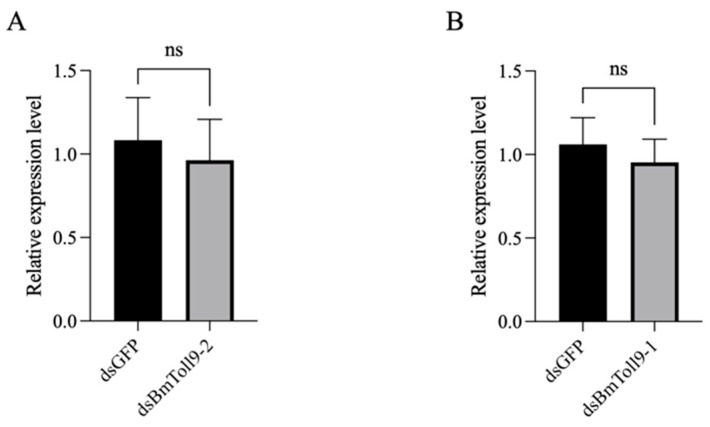
Relative expression of *BmToll9-1* and *BmToll9-2* genes silenced by each other in *B. mori* 5th instar larvae. (**A**) Relative expression of *BmToll9-1* in ds*BmToll9-2*-injected larvae. (**B**) Relative expression of *BmToll9-2* in ds*BmToll9-1*-injected larvae. Data were presented as means ± standard deviations of three biological replicates. ns—not significant.

It is worth noting that *BmToll9-2* appeared to share similar functions with *BmToll9-1*. To clarify whether these two genes affect each other’s expression, a cross-validation strategy was employed. In the *BmToll9-2*-silenced larvae, the expression of *BmToll9-1* remained unchanged compared to the control group (Figure 5A). Likewise, silencing BmToll9-1 had no discernible impact on the expression of *BmToll9-2* (Figure 5B). This analysis underscores that *BmToll9-1* and *BmToll9-2*, while functionally similar, are distinct genes with independent regulatory mechanisms.

## 4. Discussion

Previous studies have revealed that *BmToll9-1* plays a crucial role in the innate immune pathway, exhibiting distinct responses to various microbial infections and functioning as a pattern recognition receptor (PRR) for LPS [31,33,39]. Additionally, *BmToll9-1* has been confirmed to positively mediate the innate immune pathway by regulating the expression of Toll pathway signaling genes and most AMP genes [6]. In this study, we further investigated the role of *BmToll9-1* as a sensor for bacteria in modulating larval development and downstream Toll signaling pathways.

### 4.1. BmToll9-1 Might Be Involved in Immune Response to Regulate Development of the Silkworm

Previously, we had shown that *BmToll9-1* is a positive regulator in the Toll pathway and the production of AMPs [6]. In this study, most Toll signaling genes were significantly up-regulated in *BmToll9-1*-silenced *B. mori* 5th instar larvae after feeding on heat-inactivated *E. coli* and *S. aureus* (Figure 3), while most AMP genes were also significantly up-regulated (Figure 4). These results suggest that *BmToll9-1* is involved in the immune response of the silkworm. At the same time, a recovery in the growth of the silkworm was observed. Following the injection of ds*BmToll9-1*, the larvae of the silkworm exhibited a reduction in both size and weight of 38% [6]. Interestingly, when *BmToll9-1*-silenced larvae were subjected to bacteria in their food, the larvae of the silkworm gradually returned to a normal weight and size (Figure 2). Combined with the above results, it was inferred that *BmToll9-1* might be involved in immune responses to regulate the development of the silkworm.

*BmToll9-1* is mainly expressed in the midgut [6]. As an important immune organ in the insect immune system, the epithelial tissue of the midgut is in close contact with the microorganisms in the midgut, and plays an important role in regulating microbial community homeostasis. In this manner, it participates in the immune processes of the organism [6,40].

Silencing of the *BmToll9-1* gene may disrupt the balance of midgut microbial homeostasis, which in turn impacts food intake and digestion, ultimately affecting the growth and development of larvae. A similar observation was documented in our previous study concerning the *BmToll9-2* gene [34]. It has been demonstrated that the gut microbiota plays a role in the development of insects [41,42]. The relationship between immune activation and growth in silkworms likely involves complex interactions. Metabolic resources and nutrient allocation might also affect the growth of the silkworms. Future experiments should incorporate mechanistic assays, such as nutrient absorption profiling, metabolic pathway analyses, and gut physiology assessments, to directly link immune up-regulation to growth outcomes.

Bacterial treatment induced the expression of *BmToll9-1*, and the body weight and size of the silkworm larvae returned to those of the control. This suggests that the reduced growth caused by the knockdown of *BmToll9-1* is reversible. Reduced larval growth due to genetic disturbances has frequently been found in past studies, e.g., involving the *BmToll9-2* and *BmPGRP-L4* genes [34,40]. It is noteworthy that these genes are associated with the regulation of the immune pathways [43].

### 4.2. BmToll9-1 Is Preferentially Triggered by Gram-Negative Bacteria

Our recent study demonstrated that silencing *BmToll9-1* resulted in a more pronounced reduction in antibacterial activity against *E. coli* compared to *S. aureus* [6]. Specifically, the induction of AMP genes by *E. coli* following *BmToll9-1* silencing was significantly higher than that induced by *S. aureus* (Figure 4). These findings suggest that the *BmToll9-1* gene is preferentially activated by Gram-negative bacteria, such as the tested *E. coli*. Although the expression difference of *BmToll9-1* was rapidly diminished in a shorter period in response to *S. aureus* (Figure 1), this appears to reflect a suppression of the overall expression profile rather than activation. Collectively, *BmToll9-1* exhibits a preferential response to *E. coli*.

Previous research has shown that *E. coli* induces the expression of *BmToll9-1* [30]. A similar response to *E. coli* was observed in our recent functional characterization of *BmToll9-2*, a gene phylogenetically very closest to *BmToll9-1*. In that study, *E. coli* strongly induced the expression of *BmToll9-2* in silkworm larvae [34]. RNAi-mediated silencing of *BmToll9-2* led to a greater reduction in the expression of AMP genes by *E. coli* compared to *S. aureus*, together with a more significant reduction in antibacterial activity against *E. coli* [35]. Given the functional and phylogenetic similarity between *BmToll9-1* and *BmToll9-2*, it is understandable that *BmToll9-1* is also specifically activated by Gram-negative bacteria.

### 4.3. BmToll9-1 and BmToll9-2 Have Separate Functions in the Silkworm Gut

Previously, we reported that the *BmToll9-1* and *BmToll9-2* genes in silkworms likely function as positive regulators of humoral immunity [6,35]. RNAi targeting of either *BmToll9-1* or *BmToll9-2* significantly reduced the expression of Toll pathway signaling genes and downstream AMP genes. Additionally, the activation of these genes exhibited a preferential response to Gram-negative bacteria [6,35]. Phylogenetic analyses further revealed that *BmToll9-1* and *BmToll9-2* are highly homologous in terms of their evolutionary relationships, suggesting they may be involved in immune regulation through functional redundancy [34].

The results of the cross-validation experiments indicated that silencing the *BmToll9-1* gene had no effect on the expression of *BmToll9-2*, and conversely, silencing the *BmToll9-2* gene did not affect the expression of *BmToll9-1* (Figure 5). This finding suggests that, regardless of the functional and phylogenetic similarities between *BmToll9-1* and *BmToll9-2*, their regulatory pathways do not affect each other’s expression, i.e., they are independent. Combined with the structural differences observed in these genes [34], our current research supports the idea that *BmToll9-1* and *BmToll9-2* represent two different and independent inputs to regulate immunity and development. Double knockdown studies need to be performed to establish whether both genes can have synergic effects.

## 5. Conclusions

This study delved into the role of the *BmToll9-1* gene as a bacterial sensor in regulating larval development and Toll signaling pathways. Silencing of *BmToll9-1* reduced the expression of signaling genes in the Toll pathway and downstream effector genes. However, bacterial challenge following the injection of ds*BmToll9-1* reinduced the expression of *BmToll9-1*, which restored the weight and size of the silkworms. Furthermore, it was observed that *E. coli* induced a higher fold change in the expression of Toll signaling and AMP genes compared to *S. aureus*. These findings suggest that *BmToll9-1* positively regulates the Toll signaling pathway, and that it is preferentially activated by the Gram-negative bacteria *E. coli*. This study also provided preliminary evidence that *BmToll9-1* (as well as *BmToll9-2*, as previously reported) activates the Toll pathway and a nearly identical set of effector genes. These two genes do not influence each other’s expression, paving the way for further investigation of their potential synergistic functions.

## Figures and Tables

**Table 1:** List of primers used in this study.

Gene	Accession Number	Primer Sequence (5′-3′)	Amplicon Size (bp)
Primers for qRT-PCR			
*BmToll9-1*	PP496203	F: CGCAGACCGTTGAGTACATG	149
		R: CCAGACTGTCGTACCTTGGT	
*BmToll9-2*	PP716770	F: GGTTACAAGCGAACGGTAGC	80
		R: CCAAATATCCGGACTGCTGC	
*BmTIF4A*	DQ443290	F: TTCGTACTGGCTCTTCTCGT	174
		R: CAAAGTTGATAGCAATTCCCT	
*BmTIF3s4*	DQ443238	F: ACTTCAAGTTCAGGGCAGAT	110
		R: TTAATTGTTTTGTGGAGGCT	
Signaling			
*BmMyD88*	XM_028186400	F: AACGGTCACGACTCGAACTC	105
		R: TCTGCCCAGATTCTTCATCC	
*BmTube*	XM_028173146	F: GGCAGAAAGTTATGGCTTGG	82
		R: ATCCTCAAATGCTCGCTGTT	
*BmPelle*	XM_028182154	F: ACATCAAGCCGGCTAACATC	117
		R: ACCGTGAGACCTTCAGATGC	
*BmCactus*	XM_028180230	F: ACAGTCGTGCGTACATTTGG	97
		R: CAGCCTCTCCCTATCGTCAA	
*BmTollip-d*	XM_021351983	F: GACGAGTCAGTCCCTCTTGC	92
		R: GTGGCTGGTGGAATTCGTAG	
*BmTollip-v*	XM_028186930	F: TGCTACTTCTGACGGTGTGG	91
		R: AGGGCCACTTTGTGGTACTG	
*BmPellino*	XM_028184930	F: AGAGTCGCTCAGCACAACAA	95
		R: CAATGTGGCTCCACACAGAT	
*BmTRAF2*	XM_028172769	F: TCGCTCCTATGGGCATAACT	118
		R: CCGCATGTTGTGATTACTGG	
*BmECSIT*	XM_028171307	F: ATGCCGCCTTAGCTAGAATG	86
		R: GCCTTTGGGCAGTACGTCTA	
Effectors			
*BmAttacin1*	NM_001043541	F: CAGTGAACTCGGATGGAACC	97
*(BmAtt1)*		R: GGCGCTGAGTACGTTCTTGT	
*BmCecropinA*	NM_001043997	F: CCGTCATAGGGCAAGCGAAA	230
*(BmCecA)*		R: AGCAATGACTGTGGTATGTCAA	
*BmDefensin*	AB_367525	F: GTTAAGTGCGGCGTTGACTG	104
*(BmDef)*		R: TGACAGGGAAAGTGGAAGGG	
*BmGloverin1*	AB_289654	F: GCTGGGATAGAAGCATCAGC	107
*(BmGlv1)*		R: ACATCAGGCCTTCTGTGACC	
*BmMoricin*	AB_006915	F: TGTGGCAATGTCTCTGGTGT	117
*(BmMor)*		R: CTGGCGATATTGATGGCTCT	
*BmLebocin3*	NM_001126260	F: CTCGATCCAAACCGAAGGTA	105
*(BmLeb3)*		R: CGGCTGGTCAAGTCCAGTAT	
*BmEnbocin*	FJ373019	F: ACCTCGCACAACTAGTTCGG	116
*(BmEnb)*		R: CCAACAGAACAAACCCACTCG	
*BmLysozyme*	NM_001043983	F: TAACGGCTCGAAGGACTACG	103
*(BmLys)*		R: GAGGTCGGAGCACTTAACGT	
*Lysozyme-like protein*	XM_012696687	F: GTTTAATCGAGCAGGGCAGC	120
*(BmLLP3)*		R: CACCCTTGCGACCTTCTTTG	
*Phenoloxidase inhibitor*	XR_001139981	F: GGATACGTGACTGGAAATGCA	102
*(BmPOI)*		R: GTCATAATCCACGGGTTTGTCC	
*Prophenoloxidase 1*	AF_178462	F: AGTGGGAAGCCATTCTCCTT	81
*(BmPPO1)*		R: GCCAGGTTTCACTCCTTGAG	
*Nitric oxide synthase 1*	XM_012689821	F: TCATCACCACTAGCGCATCC	102
*(BmNOS1)*		R: CCTTGTCCGTTCTGTGTCCT	
Primers for dsRNA synthesis			
T7-*BmToll9-1*		F: TAATACGACTCACTATAGG	531
		ACTATAGGCACAGGTCGGGT	
		R: TAATACGACTCACTATAGG	
		TCGTTGTCCCATTCGCTGAT	
T7-*BmToll9-2*		F: TAATACGACTCACTATAGG	581
		TAGTATTCTCCCGGCTCTC	
		R: TAATACGACTCACTATAGG	
		GAAGGGTGCCTTGTGTAATC	
T7-*GFP*		F: TAATACGACTCACTATAGG	495
		TACGGCGTGCAGTGCT	
		R: TAATACGACTCACTATAGG	
		TGATCGCGCTTCTCG	

## Data Availability

The raw data supporting the conclusions of this article will be made available from the corresponding author upon reasonable request.

## References

[1] Merkling S.H., van Rij R.P. (2013). Beyond RNAi: Antiviral defense strategies in *Drosophila* and mosquito. J. Insect Physiol..

[2] Chintapalli R.T.V., Hillyer J.F. (2016). Hemolymph circulation in insect flight appendages: Physiology of the wing heart and circulatory flow in the wings of the mosquito, *Anopheles gambiae*. J. Exp. Biol..

[3] Cheng T.C., Zhang Y.L., Liu C., Xu P.Z., Gao Z.H., Xia Q.Y., Xiang Z.H. (2008). Identification and analysis of Toll-related genes in the domesticated silkworm, *Bombyx mori*. Dev. Comp. Immunol..

[4] Nüsslein-Volhard C., Lohs-Schardin M., Sander K., Cremer C. (1980). A dorso-ventral shift of embryonic primordia in a new maternal-effect mutant of *Drosophila*. Nature.

[5] Gay N.J., Keith F.J. (1991). *Drosophila* Toll and IL-1 receptor. Nature.

[6] Liu J., Chen W., Situ J., Li J., Chen J., Lai M., Huang F., Li B. (2024). BmToll9-1 is a positive regulator of the immune response in the silkworm *Bombyx mori*. Insects.

[7] Hashimoto C., Hudson K.L., Anderson K.V. (1988). The Toll gene of *Drosophila*, required for dorsal-ventral embryonic polarity, appears to encode a transmembrane protein. Cell.

[8] Valanne S., Wang J., Rämet M. (2011). The *Drosophila* Toll Signaling Pathway. J. Immunol..

[9] Nonaka S., Kawamura K., Hori A., Salim E., Fukushima K., Nakanishi Y., Kuraishi T. (2018). Characterization of Spz5 as a novel ligand for *Drosophila* Toll-1 receptor. Biochem. Biophys. Res. Commun..

[10] Chowdhury M., Li C.F., He Z., Lu Y., Liu X.S., Wang Y.F., Ip Y.T., Strand M.R., Yu X.Q. (2019). Toll family members bind multiple Spatzle proteins and activate antimicrobial peptide gene expression in *Drosophila*. J. Biol. Chem..

[11] Eldon E., Kooyer S., D’Evelyn D., Duman M., Lawinger P., Botas J., Bellen H. (1994). The *Drosophila* 18 wheeler is required for morphogenesis and has striking similarities to Toll. Development.

[12] Tauszig S., Jouanguy E., Hoffmann J.A., Imler J.-L. (2000). Toll-related receptors and the control of antimicrobial peptide expression in *Drosophila*. Proc. Natl. Acad. Sci. USA.

[13] Ooi J.Y., Yagi Y., Hu X., Ip Y.T. (2002). The *Drosophila* Toll-9 activates a constitutive antimicrobial defense. EMBO Rep..

[14] Bettencourt R., Tanji T., Yagi Y., Ip Y.T. (2004). Toll and Toll-9 in *Drosophila* innate immune response. J. Endotoxin Res..

[15] Solbakken M.H., Tørresen O.K., Nederbragt A.J., Seppola M., Gregers T.F., Jakobsen K.S., Jentoft S. (2016). Evolutionary redesign of the Atlantic cod (*Gadus morhua L*.) Toll-like receptor repertoire by gene losses and expansions. Sci. Rep..

[16] Williams M.J. (1997). The 18-wheeler mutation reveals complex antibacterial gene regulation in *Drosophila* host defense. EMBO J..

[17] Kambris Z., Hoffmann J.A., Imler J.-L., Capovilla M. (2002). Tissue and stage-specific expression of the Tolls in *Drosophila* embryos. Gene Expr. Patterns.

[18] Kolesnikov T., Beckendorf S.K. (2007). 18 Wheeler regulates apical constriction of salivary gland cells via the Rho-GTPase-signaling pathway. Dev. Biol..

[19] Seppo A., Matani P., Sharrow M., Tiemeyer M. (2003). Induction of neuron-specific glycosylation by Tollo/Toll-8, a *Drosophila* Toll-like receptor expressed in non-neural cells. Development.

[20] Kim S., Chung S., Yoon J., Choi K.W., Yim J. (2006). Ectopic expression of Tollo/Toll-8 antagonizes Dpp signaling and induces cell sorting in the *Drosophila* wing. Genesis.

[21] Aronstein K.A., Saldivar E. (2005). Characterization of a honey bee Toll related receptor gene Am18w and its potential involvement in antimicrobial immune defense. Apidologie.

[22] Christophides G.K., Zdobnov E., Barillas-Mury C., Birney E., Blandin S., Blass C., Brey P.T., Collins F.H., Danielli A., Dimopoulos G. (2002). Immunity-Related Genes and Gene Families in *Anopheles gambiae*. Science.

[23] Zou Z., Evans J.D., Lu Z., Zhao P., Williams M., Sumathipala N., Hetru C., Hultmark D., Jiang H. (2007). Comparative genomic analysis of the *Tribolium* immune system. Genome Biol..

[24] Tanaka H., Ishibashi J., Fujita K., Nakajima Y., Sagisaka A., Tomimoto K., Suzuki N., Yoshiyama M., Kaneko Y., Iwasaki T. (2008). A genome-wide analysis of genes and gene families involved in innate immunity of *Bombyx mori*. Insect Biochem. Mol. Biol..

[25] Xia Q., Zhou Z., Lu C., Cheng D., Dai F., Li B., Zhao P., Zha X., Cheng T., Chai C. (2004). A draft sequence for the genome of the domesticated silkworm (*Bombyx mori*). Science.

[26] Mita K., Kasahara M., Sasaki S., Nagayasu Y., Yamada T., Kanamori H., Namiki N., Kitagawa M., Yamashita H., Yasukochi Y. (2004). The genome sequence of silkworm, *Bombyx mori*. DNA Res..

[27] Liu J., Yang Q., Yang Y., Lin X. (2024). Larval RNA Interference in Silkworm Bombyx mori through Chitosan/dsRNA Nanoparticle Delivery. J. Vis. Exp..

[28] Yang W., Lin Y., He Y., Li Q., Chen W., Lin Q., Swevers L., Liu J. (2024). BmPGPR-L4 is a negative regulator of the humoral immune response in the silkworm *Bombyx mori*. Arch. Insect Biochem. Physiol..

[29] Liu J., Yang Y., Yang Q., Lin X., Liu Y., Li Z., Swevers L. (2025). Successful oral RNA interference efficiency in the silkworm *Bombyx mori* through nanoparticle-shielded dsRNA delivery. J. Vis. Exp. JoVE.

[30] Wu S., Zhang X., Chen X., Cao P., Beerntsen B.T., Ling E. (2010). BmToll9, an Arthropod conservative Toll, is likely involved in the local gut immune response in the silkworm, *Bombyx mori*. Dev. Comp. Immunol..

[31] Liu J., Smagghe G., Swevers L. (2013). Transcriptional response of BmToll9-1 and RNAi machinery genes to exogenous dsRNA in the midgut of *Bombyx mori*. J. Insect Physiol..

[32] Liu J., Kolliopoulou A., Smagghe G., Swevers L. (2014). Modulation of the transcriptional response of innate immune and RNAi genes upon exposure to dsRNA and LPS in silkmoth-derived Bm5 cells overexpressing BmToll9-1 receptor. J. Insect Physiol..

[33] Zhang R., Li X., Zhang J., Li Y., Wang Y., Song Y., Ren F., Yi H., Deng X., Zhong Y. (2021). Toll9 from *Bombyx mori* functions as a pattern recognition receptor that shares features with Toll-like receptor 4 from mammals. Proc. Natl. Acad. Sci. USA.

[34] Liu J., Yang W., Liao W., Huang Y., Chen W., Bu X., Huang S., Jiang W., Swevers L. (2024). Immunological function of *Bombyx* Toll9-2 in the silkworm (*Bombyx mori*) larval midgut: Activation by *Escherichia coli*/lipopolysaccharide and regulation of growth. Arch. Insect Biochem. Physiol..

[35] Liu J., Chen W., Chen S., Li S., Swevers L. (2024). Similarly to BmToll9-1, BmToll9-2 Is a Positive Regulator of the Humoral Immune Response in the Silkworm, *Bombyx mori*. Insects.

[36] Geng T., Huang Y., Hou C., Qin G., Lv D., Guo X. (2016). Inductive expression patterns of genes related to Toll signaling pathway in silkworm ( *Bombyx mori* ) upon Beauveria bassiana infection. J. Asia-Pac. Entomol..

[37] Wang G.H., Xia Q.Y., Cheng D.J., Duan J., Zhao P., Chen J., Zhu L. (2008). Reference genes identified in the silkworm *Bombyx mori* during metamorphism based on oligonucleotide microarray and confirmed by qRT-PCR. Insect Sci..

[38] Livak K.J., Schmittgen T.D. (2001). Analysis of Relative Gene Expression Data Using Real-Time Quantitative PCR and the 2−ΔΔCT Method. Methods.

[39] Wu S., Zhang X., He Y., Shuai J., Chen X., Ling E. (2010). Expression of antimicrobial peptide genes in *Bombyx mori* gut modulated by oral bacterial infection and development. Dev. Comp. Immunol..

[40] Liang Y., Wang T., Yang W., Chen Z., Li Q., Swevers L., Liu J. (2023). Silencing of the immune gene BmPGRP-L4 in the midgut affects the growth of silkworm (*Bombyx mori*) larvae. Insect Mol. Biol..

[41] Qiao H., Keesey I.W., Hansson B.S., Knaden M. (2019). Gut microbiota affects development and olfactory behavior in *Drosophila melanogaster*. J. Exp. Biol..

[42] Guo B., Tang J., Ding G., Mashilingi S.K., Huang J., An J. (2023). Gut microbiota is a potential factor in shaping phenotypic variation in larvae and adults of female bumble bees. Front. Microbiol..

[43] Bai S., Yao Z., Raza M.F., Cai Z., Zhang H. (2021). Regulatory mechanisms of microbial homeostasis in insect gut. Insect Sci..

